# Draft genome of uropathogenic *Escherichia coli* strains of B2 phylogroup

**DOI:** 10.1128/mra.00366-26

**Published:** 2026-08-27

**Authors:** Isabel Montserrat Cortez-de la Puente, Rosa del C. Rocha-Gracia, Patricia Lozano-Zaraín, Margarita M. P. Arenas-Hernández

**Affiliations:** 1 Posgrado en Microbiología, Centro de Investigación en Ciencias Microbiológicas (CICM), Instituto de Ciencias (ICUAP), Benemérita Universidad Autónoma de Puebla (BUAP), Ciudad Universitaria, Puebla, Pue, Mexico; Fluxus Inc., Sunnyvale, California, USA

**Keywords:** genome sequencing, uropathogenic *E. coli*, urinary tract infection, virulence determinants

## Abstract

Uropathogenic *Escherichia coli* (UPEC) causes urinary tract infections worldwide. We report the draft genomes of three UPEC strains from Mexican patients. The genomes ranged from 5.05 to 5.81 Mb, with 50.0–50.5% guanine-cytosine content and 4,960–6,013 predicted genes.

## ANNOUNCEMENT

Uropathogenic *Escherichia coli* (UPEC) is the main etiological agent of urinary tract infections (UTIs) (1). In Mexico, UTIs rank third among the 20 leading causes of national disease, representing a public health problem (2). The strains CR135, CR149, and 124N were isolated from urine from patients with UTI and belong to a strain collection previously characterized. The virulence factors and serotyping were previously determined by PCR and using conventional antisera techniques, respectively (Table 1) (3–7). The strains were originally isolated by plating 10 μL of urinary sample on MacConkey agar and incubated aerobically at 37°C overnight. A lactose-positive colony was selected for identification by conventional biochemical tests, including indole production, citrate utilization, and sugar fermentation (8). Additionally, the phylogenetic group of the strains was determined using the Clermont method (9). For CR135, CR149, and 124N, approval by an ethics committee was not required because the strain was donated by a clinical laboratory in Puebla (CR135 and CR149) and the Department of Public Health of the Faculty of Medicine by Universidad Autónoma de México (124N). After isolation, each strain was maintained at −70°C. For genomic DNA extraction, each strain was plated on MacConkey agar and incubated at 37°C for 24 h, and one lactose-positive colony was inoculated in 3 mL of LB broth and incubated aerobically at 37°C (160 rpm) overnight. Genomic DNA was isolated using Wizard Genomic DNA Purification Kit (Promega, USA). All protocols were conducted following the manufacturer’s instructions. The genomic DNA was resolved on 0.5% agarose gel to determine its integrity. Genomes were sequenced by Illumina technology; Illumina DNA Prep Kit (Illumina, USA) was used to prepare the libraries. IDT for Illumina Indexes with a target insert size of 460 bp was utilized as well. Paired-end sequencing was performed on two platforms: CR135 was sequenced on NextSeq 500 (2 × 150 bp), and CR149 and 124N were sequenced on MiSeq (2 × 75 bp reads). Strain CR135 has 1,189,064; CR149 has 5,603,165; and 124N has 5,263,129 paired reads. The final genome coverage was 150× for CR135 and 100× for CR149 and 124N (estimated). In the following analysis, default settings were used unless otherwise noted. For quality control and error correction of the raw reads, FastQC (v0.12.1) and trimmomatic (v0.36) were used (10, 11). *De novo* assembly of the reads was performed by SPAdes (v3.15.4) (12). Assembly quality was assessed with QUAST and CheckM2 (v1.1.0), showing 100% completeness and <1.5% contamination in all assemblies (13, 14). For CR135, CR149, and 124N, three, 206, and 91 contigs, respectively, were removed because they were shorter than 200 bp. CR135 has a genome size of 5,810,121 bp (550 contigs; 50.0% guanine-cytosine [GC]), whereas CR149 has a genome size of 5,153,506 bp (224 contigs; 50.5% GC), and 124N has a genome size of 5,050,723 bp (161 contigs; 50.5% GC). The N50 values were 26,339 bp for CR135, 224,976 bp for CR149, and 231,776 bp for 124N. Genome annotation was performed using the National Center for Biotechnology Information (NCBI Prokaryotic Genome Annotation Pipeline (PGAP) v6.6 (15), identifying 6,013 predicted genes in CR135, 5,065 in CR149, and 4,960 in 124N.

**TABLE 1:** Characteristics of three strains used in the present study and their virulence genes

Strain	Sex	Patient age	Serotyope	Virulence genes^*a*^	Reference
CR135	Female	1	O75:H5	*fimH*, *iucD*, *iha*, *sat*, *vat*, *hlyA*	(3, 4)
CR149	Female	45	O6:H1	*fimH*, *iucD*, *iha*, *sat*, *vat*, *cnf*	(3, 4)
124N	Indeterminate	Neonate	Non-typable	*fimH*, *fliC*, *satA*, *iucD*, *iha*, *vat*, *papG3*, *papG2*	(5)

^a^ Virulence genes previously determined by PCR. *fimH*, pili fimbriae adhesin type 1; *iha*, IrgA/siderophore homologous adhesin; *fliC*, flagellin; *iucD*, aerobactin; *hlyA*, α-hemolysin; *sat*, secreted autotransporter toxin; *cnf-1*, cytotoxic necrotizing factors; *traT*, serum resistance.

## Data Availability

The draft genomes of UPEC CR135, CR149, and 124N were deposited in National Center for Biotechnology Information (NCBI) under accession numbers JBSOTR000000000, JBSOTS000000000, and JBSORK000000000, respectively. In addition, all raw reads were submitted to NCBI’s SRA under accession numbers SRR36271578 to strain CR135, SRR36271672 to strain CR149, and SRR36271673 to strain 124N.
